# Emerging frontiers in phenological research

**DOI:** 10.1002/aps3.1234

**Published:** 2019-03-18

**Authors:** Elizabeth R. Ellwood, Katelin D. Pearson, Gil Nelson

**Affiliations:** ^1^ La Brea Tar Pits and Museum 5801 Wilshire Boulevard Los Angeles California 90036 USA; ^2^ California Polytechnic University 1 Grand Avenue San Luis Obispo California 93405 USA; ^3^ iDigBio Florida Museum of Natural History Gainesville Florida 32611 USA

Plant collections are, by their very nature, records of phenology—the study of cyclical events in an organism's life cycle including growth and reproduction phases that occur over the course of the year. These phases, which are called phenophases, are represented in plant collections with such features as young flower buds, senescing leaves, and even bare branches on a herbarium sheet. In combination with the collection date and locality information present on the specimen label, collections provide valuable data about plant phenology.

Phenology is not a new science, but it has taken on additional importance in recent decades as a metric for studying the impacts of global climate change on species. As the climate warms and weather patterns are altered, it becomes increasingly vital to quantify the effects of these changes on plants and animals. Certain taxa, of plants in particular, are quite sensitive to temperature, precipitation, and other environmental variables, with the timing of their growth, reproduction, and senescence advancing or becoming delayed depending on the conditions to which they are exposed.

Shifts in phenology can have cascading effects through the ecosystem. For example, the timing of when a forest leafs out signifies the start of the growing season and with it the progression of nutrient and water cycles. Likewise, if plants flower earlier in warmer springs but their insect pollinators have not yet emerged, plant and pollinator populations themselves can be negatively impacted. At the landscape scale, even small changes in phenology can have substantial consequences.

Long‐term data that capture the variability inherent to plant phenophases are foundational to phenological studies. Systematic records of phenology are rare, however, so researchers have been creative in using records that were collected for purposes other than phenological research. Herbarium specimens have proven to be especially robust sources of historic and recent phenological data, and, thanks to global efforts to image specimens and create digital records for information that was previously available only in analog format, more and more of these data are freely available online.

The urgency of biodiversity research relating to environmental change, the ready availability of phenological data, along with continually improving computational capabilities, make this an ideal time to be conducting phenological research. In this special issue, “Emerging Frontiers in Phenological Research,” nine research groups have come together to present innovative phenology projects, all of which make use of, or can be applied to, herbarium specimens.

Digitized herbarium specimens most often require the addition of phenological classification to be useful for phenological research, and this can be resource‐intensive and time‐consuming. Lorieul et al. (2019) describe advances in phenological scoring of herbarium specimens using computer neural networks, thereby streamlining the process of annotating specimens for phenophases. The authors demonstrate the usefulness of this method using plant species from numerous bioregions that exhibit a variety of phenological characteristics. Computer neural networks proved to be most accurate when annotating coarser‐scale phenophases (e.g., 50% flowering and 50% fruiting; 96.3% accurate) and slightly less accurate, although still very useful, for fine‐scale phenophases (up to 84.3% accurate). Fine‐scale phenophases may be necessary for addressing certain research questions, although as Pearson (2019) models, binary annotations (e.g., flowering/not flowering) provide nearly as much value as more precise estimations of fine‐scale phenophases. Using simulated specimen data, Pearson's models illustrate the potential to estimate specific phenophases from within the broader phenological state and the explanatory power this provides. Similarly, Ellwood et al. (2019) annotated fine‐scale reproductive and vegetative phenophases on specimens of *Acer rubrum* L. (red maple) from across eastern North America. They analyzed climate variables and combinations of these data to evaluate the effect of phenological coarseness on the strength of the models. As with Pearson, Ellwood et al. found that more precise estimates of phenophase make for slightly stronger models. However, in certain research applications, the effort required to produce accurate, fine‐scale phenophase annotations may not be worth the small statistical advantage.

To date, the majority of phenological research has involved individuals making extensive observational records, annotating herbarium specimens, and/or conducting experimental work. Most of this work has been done with temperate plant species in the Northern Hemisphere (Willis et al., 2017). A number of articles in this collection provide examples of work conducted outside of these parameters. Park et al. (2019) developed software, PhenoForecaster, that predicts the flowering times of more than 2300 angiosperm species, improving the accessibility of phenological data for use in modeling studies. PhenoForecaster greatly lowers the threshold by which a researcher can begin their phenology research, changing years of fieldwork into a relatively simple task of querying the program for instantaneous results of species flowering dates. Taxonomically understudied species are also highlighted in this collection. Andrew et al. (2019) used collections to find that fungal diversity is higher in forests than in urban areas. However, ectomycorrhizal fungal richness was found to have a positive association with tree species richness and saprotrophic fungal richness was not. Daru et al. (2019) examined a taxonomically understudied taxon, *Protea* L., in a phenologically understudied part of the world—subtropical Africa. Importantly, they found that even these botanically unique *Protea* species respond phenologically to temperature in ways that are similar to northern temperate species. This suggests that some phenology–climate relationships are generalizable across at least certain plant groups. Panchen et al.'s (2019) research investigates another geographically understudied area, the Canadian Arctic. Panchen et al. evaluate the degree to which the underrepresentation of arctic species in herbaria impacts one's ability to use collections of arctic plants for phenology research. Their findings point to spatial, temporal, and phenological biases that must be considered by researchers using these data.

Richardson et al. (2019) present a study using another transformative tool for phenological research: landscape‐scale images. Using PhenoCam images of vegetative phenology, Richardson et al. evaluated Hopkins’ Bioclimatic Law, which posits that phenology is directly related to latitude, longitude, and elevation. They find that PhenoCam data provide robust information for their analyses and that ecotypes vary in how strongly they follow the Bioclimatic Law. Integrating data from image‐based studies (e.g., Richardson et al., 2019) with data from herbarium‐based studies (e.g., Daru et al., 2019; Ellwood et al., 2019; Park et al., 2019), and even aggregating data between studies with similar data sources, can be a challenge due to the many ways phenology can be described. Fortunately, a standardized, relational vocabulary is being developed in the Plant Phenology Ontology (PPO), which enables integration of phenological data across platforms, data sources, and species (Stucky et al., 2018). Brenskelle et al. (2019) describe important updates to the PPO that will allow data from herbarium‐based studies to be represented more accurately by including descriptive terms for how measurements of plant parts relate to full plants—a relationship that was previously unaddressed in this standardized vocabulary.

This suite of articles represents work at the cutting edge of phenological research. We anticipate that these articles will provide even the most experienced phenological researcher new insights into research methods, software packages, and foundational standards and practices. Likewise, we are hopeful that this work will inspire new and experienced researchers to continue to push the boundaries of what is possible when using herbarium specimens in phenological research.

## References

[aps31234-bib-0001] Andrew, C. , U. Büntgen , S. Egli , B. Senn‐Irlet , J.‐A. Grytnes , J. Heilmann‐Clausen , L. Boddy , et al. 2019 Open‐source data reveal how collections‐based fungal diversity is sensitive to global change. Applications in Plant Sciences 7(3): e1227. 10.1002/aps3.1227 PMC6426159 30937219

[aps31234-bib-0002] Brenskelle, L. , B. J. Stucky , J. Deck , R. Walls , and R. P. Guralnick . 2019 Integrating herbarium specimen observations into global phenology data systems. Applications in Plant Sciences 7(3): e1231. 10.1002/aps3.1231 PMC6426164 30937223

[aps31234-bib-0003] Daru, B. H. , M. M. Kling , E. K. Meineke , and A. E. van Wyk . 2019 Temperature controls phenology in continuously flowering *Protea* species of subtropical Africa. Applications in Plant Sciences 7(3): e1232. 10.1002/aps3.1232 PMC6426162 30937224

[aps31234-bib-0004] Ellwood, E. R. , R. B. Primack , C. G. Willis , and J. HilleRisLamber . 2019 Phenology models using herbarium specimens are only slightly improved by using finer‐scale stages of reproduction. Applications in Plant Sciences 7(3): e1225. 10.1002/aps3.1225 PMC6426165 30937218

[aps31234-bib-0005] Lorieul, T. , K. D. Pearson , E. R. Ellwood , H. Goëau , J.‐F. Molino , P. W. Sweeney , J. M. Yost , et al. 2019 Toward a large‐scale and deep phenological stage annotation of herbarium specimens: Case studies from temperate, tropical, and equatorial floras. Applications in Plant Sciences 7(3): e1233. 10.1002/aps3.1233 PMC6426157 30937225

[aps31234-bib-0006] Panchen, Z. A. , J. Doubt , H. M. Kharouba , and M. O. Johnston . 2019 Patterns and biases in an Arctic herbarium specimen collection: Implications for phenological research. Applications in Plant Sciences 7(3): e1229. 10.1002/aps3.1229 PMC6426279 30937221

[aps31234-bib-0007] Park, I. , A. Jones , and S. J. Mazer . 2019 PhenoForecaster: A software package for the prediction of flowering phenology. Applications in Plant Sciences 7(3): e1230. 10.1002/aps3.1230 PMC6426163 30937222

[aps31234-bib-0008] Pearson, K. D. 2019 A new method and insights for estimating phenological events from herbarium specimens. Applications in Plant Sciences 7(3): e1224. 10.1002/aps3.1224 PMC6426155 30937217

[aps31234-bib-0009] Richardson, A. D. , K. Hufkens , X. Li , and T. R. Ault . 2019 Testing Hopkins’ Bioclimatic Law with PhenoCam data. Applications in Plant Sciences 7(3): e1228. 10.1002/aps3.1228 PMC6426166 30937220

[aps31234-bib-0010] Stucky, B. J. , R. Guralnick , J. Deck , E. G. Denny , K. Bolmgren , and R. Walls . 2018 The Plant Phenology Ontology: A new informatics resource for large‐scale integration of plant phenology data. Frontiers in Plant Science 9: 517. 29765382 10.3389/fpls.2018.00517 PMC5938398

[aps31234-bib-0011] Willis, C. W. , E. Law , A. C. Williams , B. F. Franzone , R. Bernardos , L. Bruno , C. Hopkins , et al. 2017 *CrowdCurio*: An online crowdsourcing platform to facilitate climate change studies using herbarium specimens. New Phytologist 215(1): 479–488. 28394023 10.1111/nph.14535

